# Anti-inflammatory and anti-oxidative effects of corilagin in a rat model of acute cholestasis

**DOI:** 10.1186/1471-230X-13-79

**Published:** 2013-05-03

**Authors:** Feng Jin, Du Cheng, Jun-Yan Tao, Shu-Ling Zhang, Ran Pang, Yuan-Jin Guo, Pian Ye, Ji-Hua Dong, Lei Zhao

**Affiliations:** 1 Department of Neurosurgery, Neuro-oncology Laboratory, Affiliated Hospital of Jining Medical College, Jining, Shandong, 272029, PR China; 2 Liver Disease Center, Department of Infectious Disease, Second Xiangya Hospital, Xiangya Medical School, Central South University, Changsha, 410011, PR China; 3 Department of Bioengineering and Therapeutic Sciences, School of Pharmacy, UCSF, CA, 94143, USA; 4 Department of Infectious Disease and Hepatology, Union Hospital, Tongji Medical College, Huazhong University of Science and Technology, Wuhan, 430022, PR China; 5 Department of Neurology, Union Hospital, Tongji Medical College, Huazhong University of Science and Technology, Wuhan, 430022, P.R. China; 6 Central Lab, Union Hospital, Tongji Medical College, Huazhong University of Science and Technology, Wuhan, 430022, P.R. China

## Abstract

**Background:**

Nowadays, treatments for cholestasis remain largely nonspecific and often ineffective. Recent studies showed that inflammatory injuries and oxidative stress occur in the liver with cholestasis. In this study, we would use corilagin to treat the animal model of acute cholestasis in order to define the activity to interfere with inflammation-related and oxidative stress pathway in cholestatic pathogenesis.

**Methods:**

Rats were administrated with alpha-naphthylisothiocyanate to establish model of cholestasis and divided into corilagin, ursodeoxycholic acid, dexamethasone, model and normal groups with treatment of related agent. At 24h, 48h and 72h time points after administration, living condition, serum markers of liver damage, pathological changes of hepatic tissue, nuclear factor (NF)-kappaB, myeloperoxidase (MPO), malondialdehyde (MDA), superoxide dismutase (SOD) and nitric oxide (NO) were examined and observed.

**Results:**

Compared to model group, corilagin had remarkable effect on living condition, pathological manifestation of liver tissue, total bilirubin, direct bilirubin, (P<0.01), but no effect on alanine aminotransferase (ALT) and aspartate aminotransferase (AST). With corilagin intervention, levels of MPO, MDA and translocation of NF-κB were notably decreased, and levels of SOD and NO were markedly increased (P<0.05 or P<0.01).

**Conclusions:**

It is shown that corilagin is a potential component to relieve cholestasis through inflammation-related and oxidation-related pathway.

## Background

Cholestasis is a reduction in bile flow that leads to the intrahepatic accumulation of bile acids and other toxic compounds with progression of liver pathology, including hepatocellular injury and fibrosis [1]. Recent studies have demonstrated that inflammatory injuries and oxidative stress occur in the liver with cholestasis [2]. Inflammatory stimulators induce signaling pathways within hepatocytes either directly, or through activation of proinflammatory cytokines, which result in suppressed expression and function of key hepatobiliary transporters and repressed expression and activity of a large number of nuclear transcriptional regulators, subsequently leading to rapid and profound reductions in bile flow [3]. This procedure enrolls neutrophils to accumulate in the liver that evoke reactive oxygen species (ROS) to produce oxidative stress and liver injury [4].

Generally in clinical practice, treatments for cholestasis remain largely nonspecific and often ineffective [5]. UDCA (ursodeoxycholic acid) is the therapeutic agent most widely used for the treatment of cholestatic hepatopathies [6]. Recent research indicated that UDCA administration early after orthotopic liver transplantation improved serum liver tests and decreased the incidence of biliary sludge and cast within the 1st postoperative year [7]. But it was concerned that further studies should be needed evaluating a longer administration of UDCA that might be even more beneficial [8]. Further, in order to obtain an effect in acute cholestasis in non-surgery condition, such as acute hepatitis, hepatic failure or drug-induced hepatic injury, UDCA should be combined with corticosteroids [9], which indicated that UDCA was a limited choice in those diseases. Another effective is glucocorticoids. It was reported that dexamethasone can decrease cholestatic liver injury within hours after bile duct ligation, which can enhance the mitochondrial biogenesis and modulate the intrinsic pathway of apoptosis following bile duct ligation [10]. But the side effects of glucocorticoids limit use in many infection or bleeding-associated diseases.

Corilagin, a member of the tannin family with its molecular formula C_27_H_22_O_18_[11], has been discovered in many medicinal plants such as Phyllanthus speices etc. [12]. Recent research indicated that corilagin has multiple activities including antioxidative, antiinflammatory, antiapoptotic, hepatoprotective and others. It was reported that corilagin could attenuate tert-butyl hydroperoxide-induced oxidative stress injury in microglial cells, which suggests that corilagin should be a potential candidate for the treatment of oxidative stress-induced neurodegenerative diseases [13]. It has been shown that corilagin has the potential to reduce HSV-1-induced inflammatory insult to the brain [14] and an anti-inflammatory activity in a cellular model [15]. Furthermore, it was confirmed that corilagin is an inhibitor of TNF-α [16] and can restrain radiation-induced microglia activation via suppression of the NF-κB pathway [17], and corilagin is protective against GalN/LPS-induced liver injury through suppression of oxidative stress and apoptosis [18]. Our recent research showed that corilagin can alleviate the hepatic fibrosis caused by egg granuloma in Schistosoma japonicum infection [19].

As nowadays there are no specific remedies for cholestasis, while corilagin can alleviate the impairment caused by inflammation and oxidation, we chose corilagin to treat the animal model of acute cholestasis in order to define the activity to interfere with inflammation-related and oxidative stress pathway in cholestasis pathogenesis.

## Methods

### Chemicals and reagents

All chemicals were purchased from Gibco (Invitrogen, city, country) or HyClone (Thermo Scientific, city, country) (like PBS and other basic stuff – this was an explanation for the authors and should not appear in the text) unless indicated otherwise. Affinity-purified rabbit anti-rat NF-κB p65 was received from Santa Cruz Biotechnology (Santa Cruz, CA). Biotin-conjugated goat anti rabbit IgG and streptavidin-horseradish peroxidase (HRP) conjugate were obtained from Kangcheng Biotech Company (Shanghai, China). Corilagin was provided by Dr. Jun-Yan Tao and friendly offered by Prof. Ji-Kai Liu, Kunming Institute of Botany, Chinese Academy of Science.

### Animals

Male Sprague–Dawley rats weighing 200–220g were purchased from the Experimental Animal Center of Tongji Medical Colllege, Huazhong University of Science and Technology. The rats were maintained under standard laboratory conditions at a temperature of 25±2°C, a relative humidity of 50±15% and normal circadian rhythm (12-h dark/12-h light). The animals were fed normal diet and water ad libitum. All study protocols were approved by internationally accepted principles and the Guidelines for the Care and Use of Laboratory Animals of Huazhong University of Science and Technology.

### Model and control establishment

90 rats were equally divided into 5 groups i.e. corilagin, UDCA, dexamethasone, model and blank control groups. Corilagin was prepared as 1.6% suspension; UDCA (Dr. Falk Pharma GmbH, Freiburg, Germany) was prepared as 0.6% suspension with water; dexamethasone (Zhejiang XianJu Pharmaceutical Company Ltd., Zhejiang, China) was dissolved in water at a concentration of 0.045%; ANIT (Sigma, St. Louis, MO) was dissolved in Sesame Oil at a concentration of 1%. Before establishing the animal model, corilagin (40 mg/kg/d), UDCA (60 mg/kg/d) and dexamethasone (1.8 mg/kg/d) were intragastrically administrated to the rats in respective group for 4 days. Model and blank control were fed by normal saline. All groups did not stop being administrated treating agent daily until executed. At the 5th day after administration and fasting for 12h, all group except normal control were intragastrically administrated ANIT (50mg/kg) for modeling. At the same day, with 8h interval the rats were still fed by respective drug or control agent. At 24h, 48h, 72h after modeling, every 6 rats in each group were executed for taking specimens. The living conditions of the victims were observed as our previous study [20].

### Specimen collection

The procedure abided by our past experiment [21]. Following anesthesia with 6% chloral hydrate by intraperitoneal injection, the rat’s abdomen was opened and abdominal aorta was separated. At the same time the common bile duct was intubated for draining bile. Then 3ml arterial blood was collected in coagulant test tube. Blood serum was obtained after 3250g centrifugation and stored at −20°C until testing. Subsequently, rat’s liver was cut by aseptic, RNase-free apparatus. After washing with normal saline, the whole hepatic tissue was divided into two parts: one was sheared and stored at −80°C, the other was fixed in 10% formalin for 48h and then dehydrated, followed by imbedding in paraffin and slicing.

### Serum markers of liver damage

The serumal total bilirubin, direct bilirubin, alanine aminotransferase (ALT) and aspartate aminotransferase (AST) were assayed by Aeroset Fully-auto Chemistry Analyzer provided by Abbott Co LTD.

### Immunohistochemistry assay

Streptavidin-perosidase (SP) immunohistochemical assay was employed to detect expression of the nuclear translocation of NF-κB. The slides of hepatic tissue were soaked in 3% H2O2-methanol solution for 20 min in order to block endogenous peroxydase. In the next 1% Triton X-100 was added at 37°C for 5min, followed by washing with PBS. After incubation with normal goat serum at room temperature for 20 min, rabbit anti-rat NF-κB p65 IgG antibody (1:200) was added dropwise and the slides were stored at 4°C overnight. The next day slides were washed with PBS and incubated with biotin-conjugated goat anti rabbit IgG for 30 min at 37°C. After another washing with PBS streptavidin-HRP was added and incubated for 30 min at 37°C. The slides were thoroughly washed with PBS 3 times for 5 min and stained with 3,3′-diaminobenzidine. Following normal dehydration, lucidification and mounting the slides were analysed under microscope (Olympus, Tokyo, Japan) as specified in our previous studies [22-25] and digital images were captured with camera (Olympus, Tokyo, Japan).

### NO and oxidates assay

Myeloperoxidase (MPO), malondialdehyde (MDA), superoxide dismutase (SOD), and nitric oxide (NO) were quantified by the respective assay kits (Nanjing Jiancheng Bioengineering Institute, Nanjing, China) according to the instructions of the manufacturer. The procedures were also described in our previous studies [26,27].

### Purity determination of corilagin by HPLC

**Figure 1:**
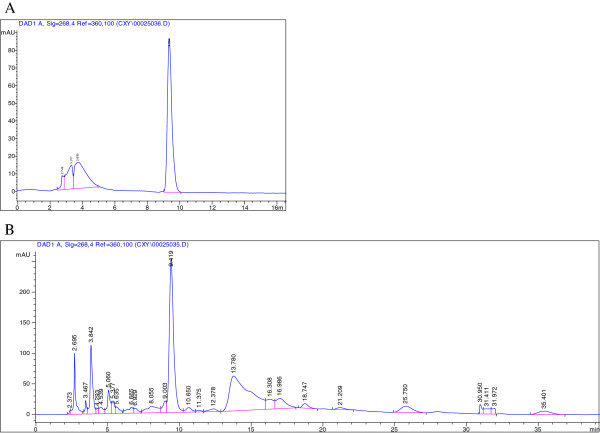
**Purity determination of corilagin by high pressure/performance liquid chromatography (HPLC). A**: The HPLC curve of corilagin standard substance; **B**: The HPLC curve of corilagin sample. Purity of corilagin was determined by HPLC. A Hanbon-Kromasil 5μm C18 column was used at 30°C with the wavelength of 268nm for detection. The mobile phase was composed of 0.5% phosphoric acid and methyl cyanide with a ratio of 76:24 and the injection volume was 10μl. The purity of corilagin was calculated to 62.14%.

Purity of corilagin was determined by HPLC. The procedure abided by our previous study [28,29]. Briefly, a Hanbon-Kromasil 5μm C18 column was used at 30°C with the wavelength of 268nm for detection. The mobile phase was composed of 0.5% phosphoric acid and methyl cyanide with a ratio of 76:24 and the injection volume was 10μl. Corilagin standard substance (purity>99%) was offered by China National Institute for the Control of Pharmaceutical and Biological Products. The purity of corilagin was calculated to 62.14%. (Figure 1)

### Statistical analysis

Data were presented as mean ± S.D. Comparisons of measurement data between multiple groups were performed with one-way ANOVA test. Comparison of the positive rate of NF-κBp65 between different groups was carried out by Pearson chi-square tests. Statistical significance was considered significant when P<0.05. Whole process was performed with SPSS 12.0 software.

## Results

### Living condition of the models

In normal group, the rats had clear urine and velvet and glossy hair. The bile could be easily drained. When taken out, the liver tissue was ruddy and lubricous. As comparison, it could be observed that the rats in model group were inactive and anorectic. Their hair was in a mess and urine was deep yellow. The drained bile decreased and their liver lost ruddy and lubricous appearance with a few of nodules at surface. The intestinal canal of the model was distended or even of toxic meteorism. Compared to model group, the rats in corilagin group appeared more active and had more appetite, more clear urine. The liver showed more ruddy and lubricous appearance with fewer nodules at surface with more easily drained bile, and the intestinal canal was less distended. The rats in UDCA group showed similar effect as corilagin group. The rats in dexamethasone group showed inactive state with proceeding weight loss and mess hair. The color of urine was clearer than that in model group, while hemorrhagic points emerged at mucous membrane in eyes, nose, month and surface of the liver. The bile in dexamethasone group could not be drained easily and the intestinal canal was dilated like that of model group.

### Serum markers of liver damage

**Figure 2:**
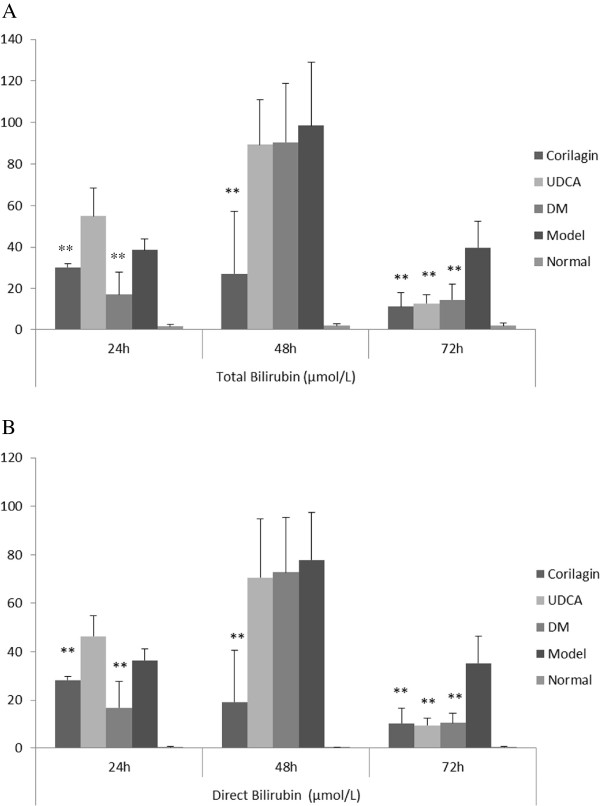
**Effect of corilagin on Liver Functional Test at 24h, 48h, 72h. A**: On Total Bilirubin; **B**: On Direct Bilirubin.The data were shown as mean±SD; ^*^p<0.05 compared to model group; ^**^p<0.01 compared to model group. When compared with model control, corilagin had a significantly decreasing effect on total bilirubin and direct bilirubin (P<0.01). At 24h, the effect of corilagin on total bilirubin and direct bilirubin was superior to that of UDCA but inferior to that of dexamethasone, while at 48h corilagin was the most effective agent (P<0.01) but at 72h the three agents had notable effect on total bilirubin and direct bilirubin level (P<0.01) . UDCA showed an effect on 72h and dexamethasone was found to have an effect on 24h and 72h on total bilirubin and direct bilirubin level (P<0.01).

As shown in Figure 2, when compared with model control, corilagin had a significantly decreasing effect on total bilirubin and direct bilirubin (P<0.01). At 24h, the effect of corilagin on total bilirubin and direct bilirubin was superior to that of UDCA but inferior to that of dexamethasone, while at 48h corilagin was the most effective agent (P<0.01) but at 72h the three agents had notable effect on total bilirubin and direct bilirubin level (P<0.01) . Corilagin had no effect on serum aminotransferase release. UDCA showed an effect on 72h and dexamethasone was found to have an effect on 24h and 72h on total bilirubin and direct bilirubin level (P<0.01).

### Pathological manifestation of hepatic tissue

**Figure 3:**
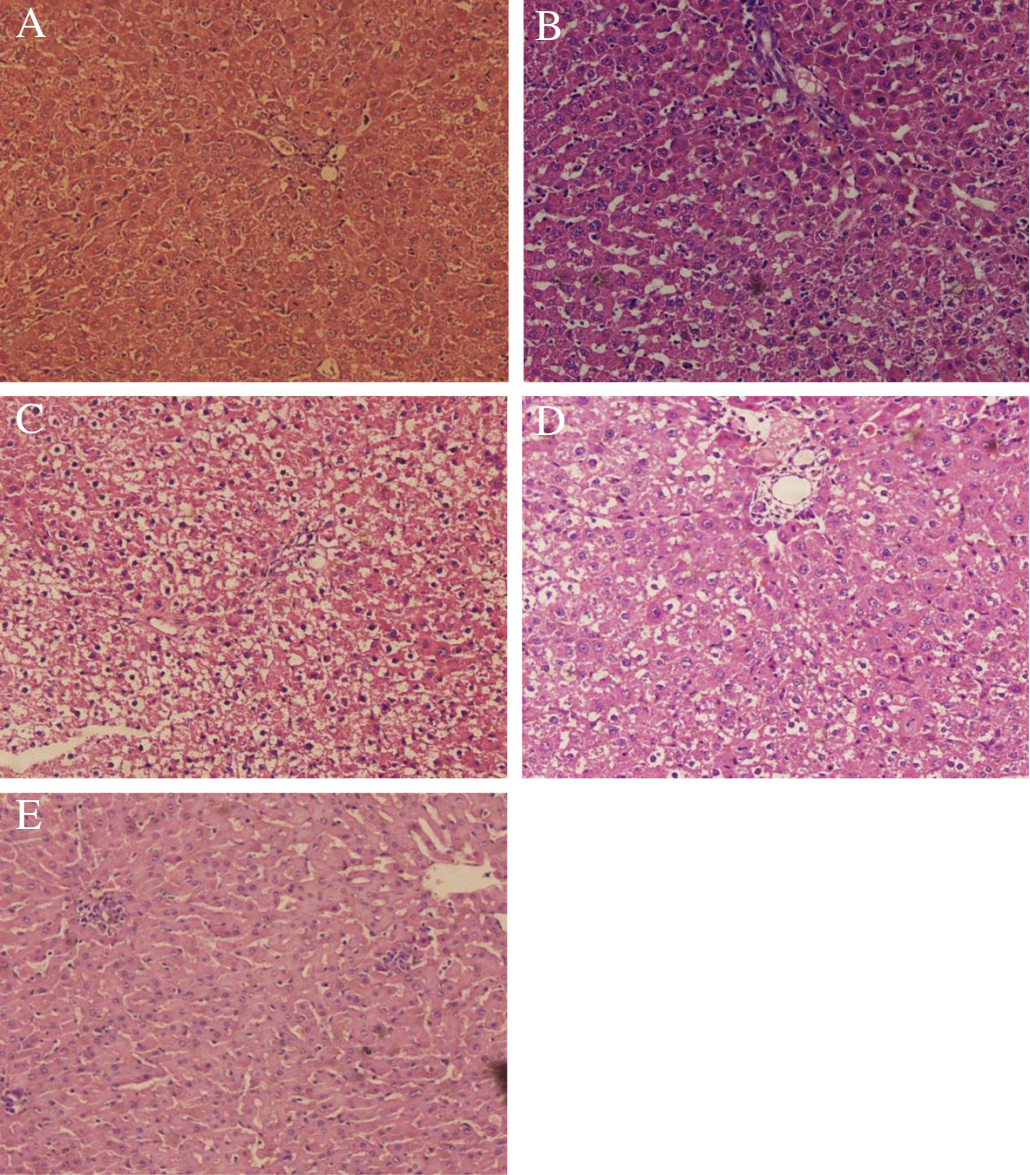
**Effect of corilagin on pathological manifestation of hepatic tissue at 48h. A**: Corilagin group; **B**: UDCA group; **C**: Dexamethasone group; **D**: Model group; **E**: Normal group. In normal group, hepatic tissue showed intact hepatic lobules, orderly liver cell cord, hepatic cell with uniform stain, epithelial cells of bile duct without damage, and no infiltration of neutrophilic granulocyte. In model group, the liver tissue showed typical pathological changes. At 48h, the liver cells showed more significantly swelling, cytoplasm with puff, ununiformed nucleus with accumulative chromatin and enlarged, strong-stained nucleolus. The liver tissue showed plenty of punctiform or focused necrotic zones and proliferation of Kupffer cells and bile duct epithelial cells all over the visual field. The bile duct exhibited a constrictive canal with necrotic cells and bile thrombus. Many necrotic hepatocytes and infiltrated neutrophils were present around the bile duct. In the corilagin group the pathological changes were significantly lower than in the model group. The manifestations in the UDCA group were a little severer than in the corilagin group while in the dexamethasone group the pathological impairment appeared more aggravated.

As shown in Figure 3, pathological features were differently presented in each group. In normal group, hepatic tissue showed intact hepatic lobules, orderly liver cell cord, hepatic cell with uniform stain, epithelial cells of bile duct without damage, and no infiltration of neutrophilic granulocyte. In model group, the liver tissue showed typical pathological changes. At 24h, destruction of hepatic lobules, hydropic degeneration or feather-like degeneration in liver cells, swelling amotic bile duct epithelial cell and infiltration of neutrophilic granulocyte in portal area were observed. At 48h, the liver cells showed more significantly swelling, cytoplasm with puff, ununiformed nucleus with accumulative chromatin and enlarged, strong-stained nucleolus. The liver tissue showed plenty of punctiform or focused necrotic zones and proliferation of Kupffer cells and bile duct epithelial cells all over the visual field. The bile duct exhibited a constrictive canal with necrotic cells and bile thrombus. Many necrotic hepatocytes and infiltrated neutrophils were present around the bile duct. At 72h, the pathological changes were recovered slightly but still featured with necrotic regions and neutrophil infiltration. In the corilagin group the pathological changes were significantly lower than in the model group. The manifestations in the UDCA group were a little severer than in the corilagin group while in the dexamethasone group the pathological impairment appeared more aggravated.

### Expression of NF-κBp65 examined by immunohistochemistry

**Figure 4:**
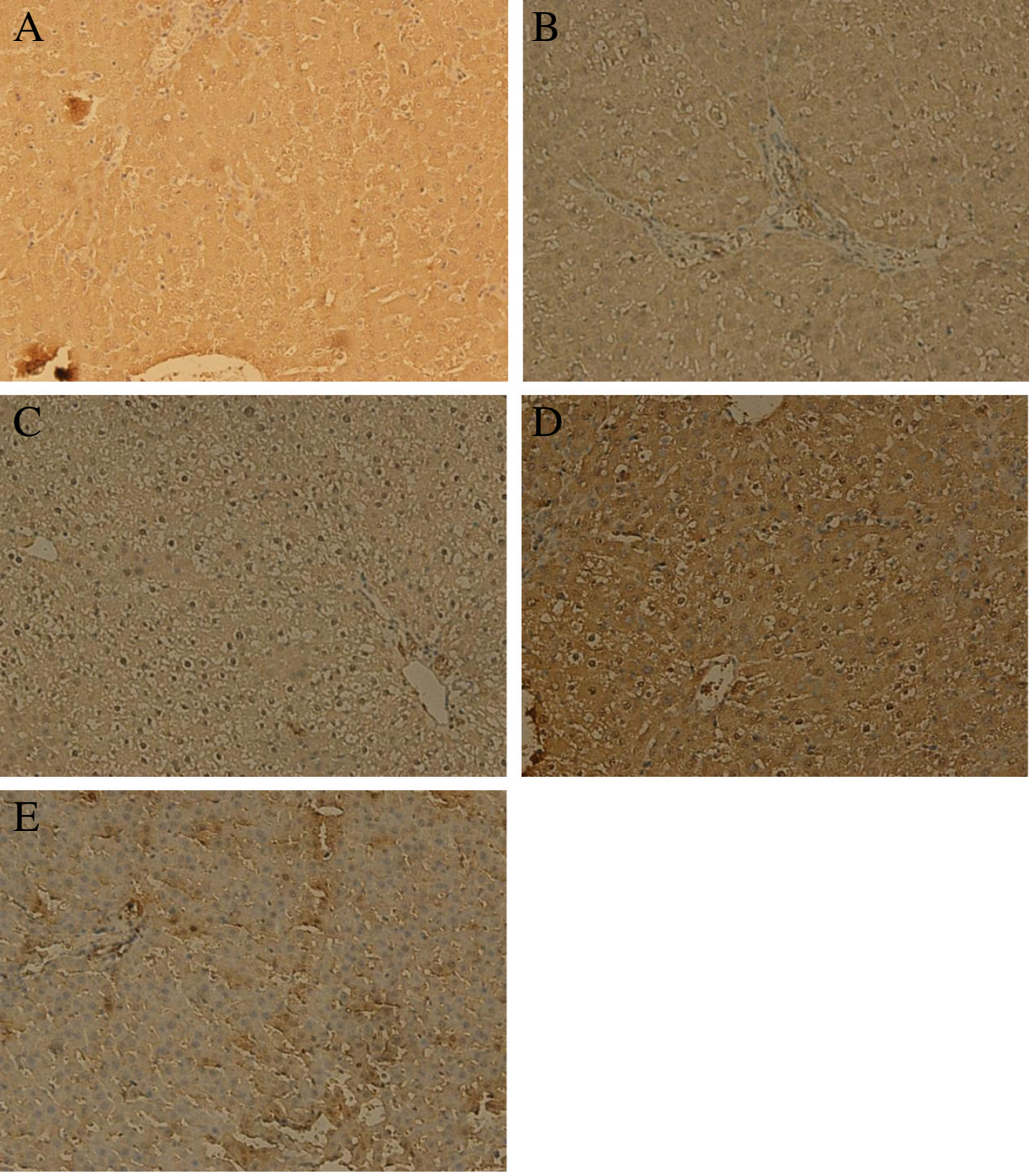
**Effect of corilagin on NF-κBp65 expression at 48h. The activated NF-κBp65 presented brown stain in nuclear and inactivated NF-κBp65 presented brown in cytoplasm. A**: Corilagin group; **B**: UDCA group; **C**: Dexamethasone group. **D**: Model group; **E**: Normal group. It was shown that in normal group the staining of NF-κBp65 in nucleus was not remarkable. After ANIT administration, the positive rate of NF-κBp65 staining in nucleus significantly rose (P<0.01). With corilagin treatment, the rate of NF-κBp65 staining in nucleus was decreased markedly (P<0.01). In UDCA and dexamethasone group the rate was also significantly lower than that in model group but higher than that in corilagin group (P<0.05).

As shown in Figure 4, it was shown that in normal group the staining of NF-κBp65 in nucleus was not remarkable. After ANIT administration, the positive rate of NF-κBp65 staining in nucleus significantly rose (P<0.01). With corilagin treatment, the rate of NF-κBp65 staining in nucleus was decreased markedly (P<0.01). In UDCA and dexamethasone group the rate was also significantly lower than that in model group but higher than that in corilagin group (P<0.05).

### Levels of MPO, MDA, SOD and NO in tissue

**Figure 5:**
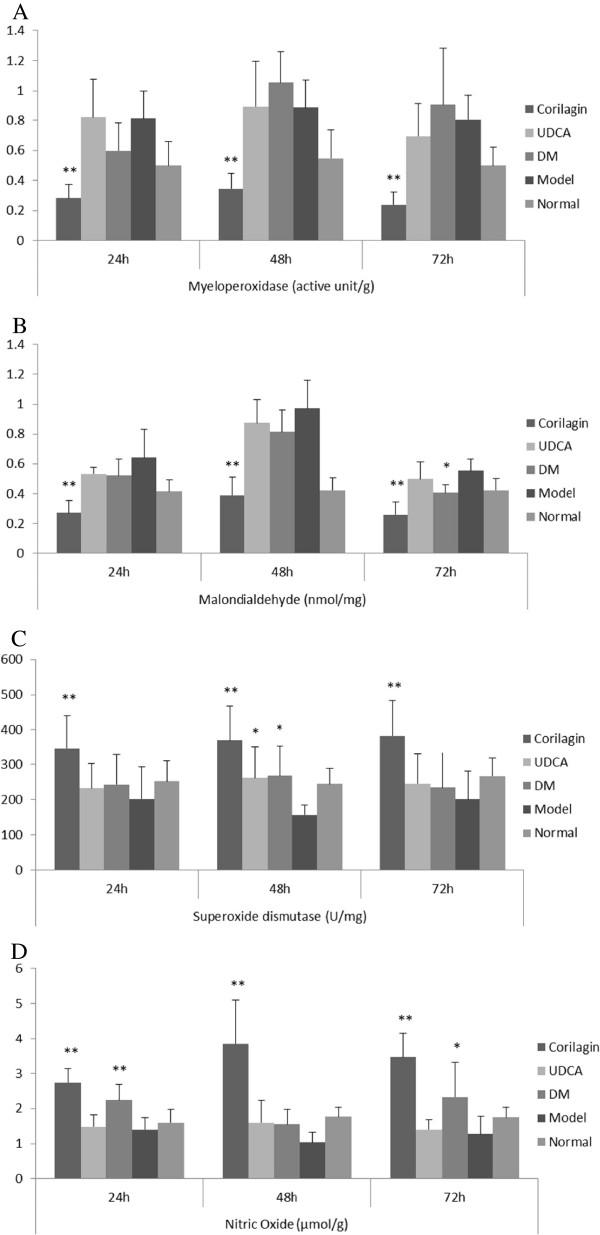
**Effect of corilagin on anti-oxidative and nitric oxide at 24h, 48h, 72h. A**: On myeloperoxidase; **B**: On malondialdehyde; **C**: On superoxide dismutase; **D**: On nitric oxide. The data were shown as mean±SD; ^*^p<0.05 compared to model group; ^**^p<0.01 compared to model group. The level of MPO and MDA significantly rose in the model group (P<0.01). At all-time points the levels of the two enzymes were notably down-regulated by corilagin treatment and even lower than the normal level. As control, UDCA had no similar effect as corilagin, and dexamethasone only had an effect on MDA at 72h (P<0.05). In contrast, the levels of SOD and NO in tissue decreased notably after ANIT stimulation (P<0.05 or P<0.01, respectively), while in the corilagin group the levels of SOD and NO were remarkably elevated (P<0.05 or P<0.01, respectively). UDCA had not the same effects except for SOD at 48h. Dexamethasone had an up-regulating effect on SOD at 48h, and on NO at 24h and 72h (P<0.05 or P<0.01, respectively).

As shown in Figure 5, the level of MPO and MDA significantly rose in the model group (P<0.01). At all-time points the levels of the two enzymes were notably down-regulated by corilagin treatment and even lower than the normal level. As control, UDCA had no similar effect as corilagin, and dexamethasone only had an effect on MDA at 72h (P<0.05). In contrast, the levels of SOD and NO in tissue decreased notably after ANIT stimulation (P<0.05 or P<0.01, respectively), while in the corilagin group the levels of SOD and NO were remarkably elevated (P<0.05 or P<0.01, respectively). UDCA had not the same effects except for SOD at 48h. Dexamethasone had an up-regulating effect on SOD at 48h, and on NO at 24h and 72h (P<0.05 or P<0.01, respectively).

## Discussion

Cholestasis is characterized by impaired bile flow, reduction of bile acids in the intestine, and retention of bile acids in the liver. Rats taken in alpha naphthylisothiocyanate (ANIT) have been one of the most common experimental models of intrahepatic cholestasis and used extensively, which was permitted to describe not only cholestatic alterations but also compensatory mechanisms [30]. The liver in ANIT-treated rats showed cholangiolitic hepatitis characterized by intrahepatic cholestasis, necrosis of hepatocytes and biliary epithelial cells and bile obstruction [31].

In current clinical practice, initial assessment of hepatobiliary diseases is accomplished by measuring serum concentrations of bile acids and bilirubin as well as serum activities of liver-associated enzymes which reveal information about the state of liver. Our previous study showed that the indexes of liver damage and pathological changes start to rise at 24h after ANIT treatment, reach a maximum at 48h and trend to restore at 72h [21]. The data of the present study imply that the most effective change after corilagin treatment refers to bilirubin, both total and direct. Compared to UDCA and dexamethasone, corilagin showed a protective effect on the elevation of serum bilirubin after hepatic injury. However, there is no obvious evidence of corilagin to alleviate aminotransferases, which indicates that corilagin has a stronger effect on relieving bile or bile duct disorders.

The ANIT hepatotoxicity is attributed to both glutathione and blood neutrophils. Glutathione can form a reversible S-conjugate with ANIT that is critical in shuttling ANIT into bile, where it induces a group of toxic substances[32], and neutrophils may be activated during ANIT exposure to release cytotoxic proteases that cause injury to target cells [33]. In our study, with ANIT treatment the rat liver showed typical damage of neutrophilic infiltration, necrosis of hepatocytes, proliferation of inflammatory cells and epithelial cells of bile duct and formation of bile thrombus. By corilagin intervention, it could be observed that improvement of the acute hepatic impairment was achieved, which shows the protective effect of corilagin on hepatic pathology.

ANIT-evoked hepatotoxins cause severe neutrophilic inflammation around portal tracts and bile ducts and induce significant inflammatory reactions in the liver, which suggests that the inflammation plays a central role in cholestatic hepatitis. Nuclear factor-kappa B (NF-κB) is a pivotal factor that transfers inflammatory signals from cytoplasm into the nucleus and induces a series of inflammatory responses in the cell [34,35]. In our present study, it was found that NF-κB was notably suppressed in the corilagin group, which demonstrates that corilagin has the efficacy to control inflammatory injuries during cholestasis in the liver.

The reactive oxygen species are generated by aerobic metabolism and environmental stressors. They can chemically modify proteins and alter their biological functions and, if the repair processes fail, oxidized proteins may become cytotoxic [36]. Myeloperoxidase (MPO), a heme-containing peroxidase abundantly expressed in neutrophils and monocytes, while produces the powerful oxidant hypochlorous acid and is a key contributor to the oxygen-dependent microbicidal activity of phagocytes, is linked to tissue damage in many diseases when it is excessive to generate MPO-derived oxidants [37]. In our research, corilagin displayed a strong effect on MPO, demonstrating the oxidative impairment of neutrophils can be prevented by corilagin. Malondialdehyde (MDA) is the principal and most studied product of polyunsaturated fatty acid peroxidation. So far, oxidative stress have been assessed by measuring the level of antioxidants or the concentration of substances derived from the action of oxygen free radicals on biological molecules, and measurement of MDA is considered an effective marker of oxidative stress in a biological sample [38]. In our study, it could be showed that corilagin has a significant effect on MDA expression, which implies corilagin has a strong potential to suppress oxidative stress when cholestasis occurs. After oxidative stress occurs, products of lipid peroxidation created in different biochemical reactions are normally removed by antioxidants, the compounds that are involved in effective scavenging of free radicals and in suppressing the actions of reactive oxygen substances [39]. The most important enzymatic antioxidants are superoxide dismutase (SOD), a sort of enzyme that catalyze the dismutation of superoxide radical into hydrogen peroxide (H2O2) and molecular oxygen (O2) and consequently present an important defense mechanism against superoxide radical toxicity [40]. In the present research, the SOD level in corilagin group was markedly higher than that in model group, which showed the anti-oxidative effect of corilagin.

Nitric oxide (NO) plays a critical role on hepatic metabolism whether liver is in normal condition or in injury by different agents. Although some experiments show controversial effect of NO in the liver on pathological condition, literature from the past 15 years seems to reinforce the consensus that NO is indeed protective. Some of the protective actions of NO are due to its potential as an antioxidant and anti-inflammatory agent, along with its beneficial effects on cell signaling and inhibition of nuclear proteins, such as NF-kappa B and AP-1, especially when ischemia occurs [41,42]. The supplementation with a NO donor prevented caspase-3 activity and apoptosis induced by bile acids in cultured rat hepatocytes [43]. In our experiment, after corilagin treatment the expression of NO rose notably, which implies corilagin can improve circulation in the liver.

Corilagin has been found in many medicinal plants and some advanced technologies have been employed to detect the content of corilagin in those plants [44]. Although corilagin can be synthesized by chemicals, the actual output for synthesis is limited [11]. Thereby some novel techniques were used to purify corilagin from plants [45]. In our research, we identified the content of corilagin in extraction and the purity of corilagin is dominant in the extraction.

## Conclusions

In conclusion, we found that corilagin has the capability to ensure hepatic protection, to block NF-κB pathway, to provide anti-oxidative effects and to improve hepatic circulation in experimental intrahepatic cholestasis, which suggests that corilagin is a putative medication to treat cholestasis. It is shown that corilagin has the potential to cure inflammation-related and oxidation-related diseases. The further proceeding might be to investigate how corilagin interacts with the inflammatory and oxidative signal pathways in cell and animal models.

## Competing interests

The authors declare that they have no competing interests.

## Authors’ contributions

FJ participated in the serum markers of liver damage and drafted the manuscript. DC took part in immunohistochemistry. JY Tao carried out purity determination of corilagin. RP and YGparticipated in detection of oxidation-related enzymes. PY performed animal feeding and observation. JD and SZ participated in the design of the study and performed the statistical analysis. LZ conceived of the study, and participated in its design and coordination. All authors read and approved the final manuscript.

## Pre-publication history

The pre-publication history for this paper can be accessed here:

http://www.biomedcentral.com/1471-230X/13/79/prepub
